# Utilizing adaptive deformable convolution and position embedding for colon polyp segmentation with a visual transformer

**DOI:** 10.1038/s41598-024-57993-0

**Published:** 2024-03-27

**Authors:** Mohamed Yacin Sikkandar, Sankar Ganesh Sundaram, Ahmad Alassaf, Ibrahim AlMohimeed, Khalid Alhussaini, Adham Aleid, Salem Ali Alolayan, P. Ramkumar, Meshal Khalaf Almutairi, S. Sabarunisha Begum

**Affiliations:** 1 Department of Medical Equipment Technology, College of Applied Medical Sciences, Majmaah University, Al Majmaah, 11952 Saudi Arabia; 2 Department of Artificial Intelligence and Data Science, KPR Institute of Engineering and Technology, Coimbatore, 641407 India; 3 Department of Biomedical Technology, College of Applied Medical Sciences, King Saud University, Riyadh, 12372 Saudi Arabia; 4 Department of Computer Science and Engineering, Sri Sairam College of Engineering, Anekal, Bengaluru, 562106 Karnataka India; 5 Department of Biotechnology, P.S.R. Engineering College, Sivakasi, 626140 India

**Keywords:** Polyp segmentation, Vision transformer, Deformable convolution, Engineering, Biomedical engineering

## Abstract

Polyp detection is a challenging task in the diagnosis of Colorectal Cancer (CRC), and it demands clinical expertise due to the diverse nature of polyps. The recent years have witnessed the development of automated polyp detection systems to assist the experts in early diagnosis, considerably reducing the time consumption and diagnostic errors. In automated CRC diagnosis, polyp segmentation is an important step which is carried out with deep learning segmentation models. Recently, Vision Transformers (ViT) are slowly replacing these models due to their ability to capture long range dependencies among image patches. However, the existing ViTs for polyp do not harness the inherent self-attention abilities and incorporate complex attention mechanisms. This paper presents Polyp-Vision Transformer (Polyp-ViT), a novel Transformer model based on the conventional Transformer architecture, which is enhanced with adaptive mechanisms for feature extraction and positional embedding. Polyp-ViT is tested on the Kvasir-seg and CVC-Clinic DB Datasets achieving segmentation accuracies of 0.9891 ± 0.01 and 0.9875 ± 0.71 respectively, outperforming state-of-the-art models. Polyp-ViT is a prospective tool for polyp segmentation which can be adapted to other medical image segmentation tasks as well due to its ability to generalize well.

## Introduction

Colon is a gastrointestinal tract, consisting of the colon, rectum, and anus. Polyp is a kind of abnormal protuberance in the colon. Colonic polyps are usually benign and are usually not cancerous, but they can be precursors of cancer. CRC is a type of cancer in which malignant cells develop from the colon tissue. CRC is identified to be the third most common kind of cancer worldwide and second major cause of death due to cancer^1^. Colonoscopy is an invasive procedure, which is used to examine the inside of the colon for polyps and other abnormalities.

In the last few years, several methods have been developed to automate polyp detection. Kudo et al.^2^ have presented an extensive review on artificial intelligence-based models^3^ for polyp detection and characterization and advocate the need for getting regulatory approvals for using them in real clinical settings. Deep learning models have gained significant attention due to their ability to learn intrinsic features, discern subtle differences and perform high-level reasoning from data. Recently deep learning models for polyp classification^4^, detection^5,6^ and segmentation^7,8^ have been proposed towards accurate and robust detection of polyps. Polyps appear as elongated protrusions of colonic mucosa. They are isolated or clustered lumps on the colonic wall, with heterogeneous morphologies. They are usually small, but may grow to a size of several centimeters, with irregular surfaces, different colors and textures. The shape of a polyp can vary from spherical to cylindrical to irregular. A polyp can be either pedunculated or sessile.

While a pedunculated polyp has a stalk attached to the colon wall, a sessile polyp does not have a stalk. Further, the boundary between the polyp and the colonic wall is not well defined. Polyp segmentation is a key step in the colonic polyp detection pipeline. It is used to extract the polyp region from the colonic wall using the polyp features such as shape, color, texture and size. Segmentation of polyps is a challenging task due to the large variations in polyp shape and color, and the presence of a wide variety of other objects, such as stool, folds, and blood vessels, in the colon.

Existing deep learning models for polyp segmentation are mainly based on fully convolutional networks^9,10^. These models lack the ability to model the spatial structure of polyps and thus do not leverage the inter-polyp context information. They incorporate attention mechanisms to capture the spatial structure of polyps. In the evolving landscape of artificial intelligence applications, the use of Transformer^11–13^ models, renowned for their self-attention^14^ mechanisms, has extended its reach into the realms of agriculture and civil engineering, demonstrating their versatility and power in handling complex visual tasks. The study by Pacal et al.^15^ showcases how an advanced vision Transformer model can significantly enhance crop productivity and sustainability by accurately identifying diseases in maize leaves. By exploiting a large dataset, this application underscores the Transformer's ability to analyze extensive agricultural imagery, pinpointing disease manifestations across the leaf surface, which are crucial for timely intervention and crop management.

Similarly, the work by Guo et al.^16^ in pavement crack detection illustrates the Transformer model's effectiveness in civil engineering, specifically in maintaining infrastructure health. Their approach leverages a transformer network to meticulously analyze high-resolution pavement images, identifying cracks that vary widely in size, shape, and severity. This application highlights the model's capacity to focus on relevant spatial features and understand the global context within the images, a critical aspect in automating and enhancing the efficiency of infrastructure inspection and maintenance.

These studies exemplify the transformative impact of Transformer models in sectors beyond their initial deployment in NLP, demonstrating their adaptability and effectiveness in addressing diverse challenges in computer vision. By leveraging self-attention mechanisms to process and analyze complex patterns in data, Transformers are paving the way for innovative solutions across various domains, from boosting agricultural productivity through precise disease detection to improving infrastructure resilience via advanced crack identification techniques. Like Recurrent Neural Networks (RNNs), they are trained to process sequential data, and the attention mechanism can capture the context of a given input.

In this line, Transformer networks such as the ColonFormer^17^, SSFormer^18^ and FCN-Transformer^19^ have been successfully employed in colon polyp segmentation. However, these models are complex due to the additional attention mechanisms incorporated in them. Further, they use the conventional convolutional filters for extracting the image features, which are not suitable for the diverse morphologies of the polyps. Inspired by the ViT models proposed in^17–19^, this paper proposes a novel polyp segmentation Transformer network called Polyp Vision Transformer (Polyp-ViT), which is a dense prediction model based on deformable attention mechanism. The contributions of this research are as below.

1. The development of an Adaptive Deformable Convolutional Network (ADCN) that dynamically adjusts to the unique shapes and sizes of colon polyps, improving the precision of feature extraction.
2. The introduction of Conditional Positional Encoding (CPE) that ensures the model accurately maintains spatial relationships within images, addressing a common challenge in vision-based Transformer models.
3. The integration of a comprehensive encoder-decoder framework that combines adaptive feature extraction with global contextual understanding, enhancing segmentation accuracy.
4. An extensive evaluation on two major datasets, demonstrating improved performance in colon polyp segmentation over existing models.

The rest of this paper is organized below. A comprehensive review on deep learning models for polyp detection and segmentation is presented in related work. The proposed model, experimental results, comparative analyses, and conclusions are presented.

## Related works

This section presents a comprehensive review on automated polyp detection and segmentation models. A pilot study on the classification of polyps by Tischendorf et al.^20^ in 2010, presented a polyp classification model based on segmentation of vascular patterns and feature extraction, which achieved a classification rate of 91.9%. However, the authors identified that polyp detection by experts was superior to this model and suggested further research to improve the performance of automated models. Since the evolution of deep learning techniques, several Convolutional Neural Network (CNN) based polyp detection and segmentation models have been proposed.

In one of the pioneering works, Tajbakhsh et al.^21^ have proposed 3-way representation of images and an ensemble of CNNs for polyp detection. The color and texture, shape and temporal features are extracted at multiple scales for representation of polyp candidates using 3-channel patches. Three CNNs are trained with these features separately for polyp detection and the outputs of these classifiers are aggregated to detect the presence of polyps. This model demonstrated a highest sensitivity of 60% compared to earlier polyp detection models. Brandao et al.^22^ proposed three Fully Convolutional Neural networks (FCN) by modifying the pre-trained AlexNet^23^, GoogLeNet^24^ and VGG^25^ classifiers, for polyp segmentation. These networks are built by replacing the fully connected layers with a 1 × 1 convolutional layer. The FCN-VGG achieves the highest precision and sensitivity values of 73.61% and 86.31% respectively.

Qadir et al.^26^ proposed the Mask R-CNN framework which employs three variants of the ResNet^27^ for feature extraction for polyp segmentation. A Region Proposal Network (RPN) is trained to select the feature maps from these networks and constructs proposals or anchors at multiple resolutions and scales. The authors proposed an ensemble of two Mask-RNNs to combine the features extracted by two feature extractors, where one Mask-RNN acts as the main and the other as an auxiliary model. This model with a Resnet101 feature extractor achieves the highest Dice and Jaccard Coefficient (JC) values of 70.42% and 61.24% respectively on the MICCAI 2015 polyp detection dataset. The authors have inferred that segmentation performance can be considerably improved by training deep learning models with a better training dataset rather than using very deep and complex architectures. In segmentation models, attention mechanisms are widely used to aggregate features across multiple scales, and the attention maps are usually used as input to the subsequent layers to guide the model to focus on the most relevant part of the input image. These mechanisms are used with UNet and other segmentation models in polyp segmentation for improved performance^28–30^.

The Parallel Reverse Attention Network (Pranet)^31^ constructs a global attention map from the high-level features aggregated by a Parallel Partial Decoder (PPD). It also employs a Reverse Attention (RA) mechanism to model the polyp boundaries. Compared to the UNet and other variants, Pranet achieves the highest Dice value of 0.898 and 0.899 on the Kvasir-seg^32^ and CVC-612^33^ datasets. However, the attention maps are biased by the initial model parameters, which may not be optimal for the task at hand.

Recently, ViT models which learn the relationship between image tokens are used for attention-based segmentation. The model learns a weighted attention distribution over the input image, which is more accurate than the fixed-size attention map of the baseline model. A Transformer follows an encoder-decoder architecture with self-attention and cross-attention mechanisms to capture the long-range dependencies between image patches, exploiting the positional embeddings^34^. The Polyp-pvt^35^ is a polyp segmentation framework based on pyramid vision transformer for feature extraction and it employs three different modules for distinct tasks.

A Cascaded Fusion Module (CFM) is used to capture semantic and location information from high-level features, an attention mechanism is used to capture polyp cues from low-level features and an aggregation module to combine local and global polyp features. The effectiveness of each module is evaluated with ablation studies. This model is tested on five public datasets and achieves a highest mean Dice value of 0.937 on the ClinicDB polyp segmentation dataset. However, this model makes erroneous polyp segmentation in the presence of light, shadow and reflective points. SwinE-Net^36^ is a polyp segmentation model based on EfficientNet and a Swin Transformer network, which employs three different mechanisms for improved segmentation results. Initially, separate feature maps are extracted from the colonoscopy image using the EfficientNet and Swin Transformer.

Multi-level features are extracted from these feature maps using dilation convolutional blocks with kernels of different sizes, and a multi-feature aggregation block is used to aggregate these features to construct two initial segmentation maps. The final segmentation map is constructed by combining these maps with an attentive deconvolution network. The SwinE-Net achieves mean Dice values of 0.920 and 0.938, and mean Intersection over Union (IoU) values of 0.870 and 0.892 on the Kvasir-seg and ClinicDB datasets respectively.

The ColonFormer^17^ is hybrid polyp segmentation model based on encoder-decoder architecture, built from a hierarchical Transformer which acts as an encoder, and a hierarchical pyramid CNN which acts as a decoder. This model uses the Mix Transformer (MiT)^37^, capable of representation of coarse and fine features in the encoder. A Pyramid Pooling Model (PPM) is used to construct a global feature map from the hierarchical features extracted by the encoder. The decoder aggregates this global map with the multi-scale features extracted by the MiT and a refinement module is used to refine the boundaries of the polyps for accurate segmentation. This model achieves mean Dice values of 0.927 and 0.932, and mean IoU values of 0.877 and 0.883 on the Kvasir-seg and ClinicDB datasets respectively.

The SSFormer^18^ is a polyp segmentation model which employs an encoder implemented with PVTv2^38^, a pyramid vision transformer. The PVTv2 comprises a convolutional feed forward network with an attention layer and a patch embedding mechanism. Unlike the conventional Transformer models, it does not require explicit position embedding. The SSFormer also employs the Progressive Locality Decoder (PLD) comprising a Local Emphasis (LE) mechanism to improve the attention on polyp features and a Stepwise Feature Aggregation (SFA) module to fuse the features at multiple scales. This model achieves mean Dice values of 0.9357 and 0.9447, and mean IoU values of 0.8905 and 0.8995 on the Kvasir-seg and ClinicDB datasets respectively.

Similarly, the FCN-Transformer^19^ for polyp segmentation is a hybrid model which is implemented with a Transformer Branch (TB) for feature extraction and a Fully Convolutional Branch (FCB) for segmentation. The TB of this model is influenced by the SSFormer architecture in which, the PLD is enhanced with residual blocks, residual connections and the Sigmoid-Weighted Linear Unit (SiLU) activation function. The FCB block comprises residual blocks, convolutional layers, nearest neighbour interpolation operations and residual connections.

The FCN-Transformer is designed to fuse the coarse features extracted by the TB with the fine features extracted by the FCB to generate the segmentation maps. This model achieves mean Dice values of 0.9385 and 0.9469, and mean IoU values of 0.8903 and 0.9020 on the Kvasir-seg and ClinicDB datasets respectively. Guo et al.^39^ presented a novel polyp segmentation model called Parallel-Enhanced Network (PENet) that combines Transformer Inception (TI) and Local-Detail Augmentation (LDA) modules. The TI module enriches input features with long-range information in multiple scales using parallel Transformers with different reception fields. The LDA module applies spatial and channel attentions in parallel to enhance object details in a coarse-to-fine manner. PENet framework demonstrates efficient and accurate polyp segmentation with a mean Dice value of 0.939 on the CVC-ClinicDB dataset, outperforming state-of-the-art methods.

The Feature Decoupled Network FeDNet^40^ model for polyp segmentation improves the performance by optimizing both global contextual information and edge information simultaneously. Inspired by the feature decoupled method in Laplacian pyramid, FeDNet decouples the input feature into body and edge features using the Feature Decoupled Module (FDM). With only 0.08 m network parameters, FeDNet outperforms state-of-the-art methods, achieving a mean Dice score of 0.924 on the Kvasir-seg dataset.

The Fu-TransHNet^41^ is a new hybrid network designed for enhanced colonic polyp segmentation. The network is based on the fusion of Transformer and CNN branches, and it incorporates global and local feature learning. The network also utilizes multi-view cooperative learning techniques to determine weights and make comprehensive decisions. Experimental results demonstrate the superior performance of Fu-TransHNet compared to existing methods on various benchmark datasets, achieving notably higher mDice scores, particularly on the ETIS-LaribPolypDB^42^ dataset.

In the recent years, the following works explored the development, testing, and refinement of AI-driven tools and models aimed at improving the detection accuracy and efficiency of gastroenterological diagnostics, with a particular focus on colonoscopies. The introduction of a semi-automated video annotation tool by Krenzer et al.^43^, aimed at streamlining the machine learning annotation process for medical professionals, was foundational for the training of accurate AI systems. This was complemented by the development of a benchmark dataset, ENDOTEST, by Fitting et al.^44^, designed to rigorously evaluate computer-aided polyp detection systems, and the efforts of Brand et al.^45,46^ to develop, evaluate, and analyze the effectiveness of deep learning models and commercially available AI systems in real-world clinical settings were highlighted, showcasing the practical applications and challenges of integrating AI into current medical practices.

Further advancements were presented by Krenzer et al.^47^, where a real-time polyp detection system utilizing deep convolutional neural networks for clinical application in colonoscopy was developed, showing significant improvements in detection rates and operational efficiency. This development was seen as a crucial step towards the real-time, clinical application of AI in endoscopic procedures, potentially transforming patient outcomes through earlier and more accurate polyp detection. In an extended study, Krenzer et al.^48^ also explored the use of deep learning and Few-Shot Learning (FSL) for efficiently classifying polyps with limited data. This innovative approach demonstrates that it's possible to achieve high accuracy in polyp classification despite the scarcity of annotated images, leveraging the strengths of FSL to overcome data limitations.

Meanwhile, the optimization of polyp detection systems through the use of You Only Look Once (YOLO) algorithms, adjusted with Artificial Bee Colony (ABC) optimization techniques for enhanced detection performance, was explored by Karaman et al.^49^. The importance of optimizing AI models for specific medical applications was underscored, along with the ongoing search for more robust, real-time detection systems capable of operating efficiently under diverse and challenging conditions encountered during endoscopic procedures.

Introduced by Liu et al.^50^ was CAFE-Net, an innovative AI model that leverages cross-attention mechanisms and feature exploration networks for polyp segmentation, representing the forefront of AI research aimed at improving the granularity and accuracy of polyp detection. The employment of attention mechanisms and a focus on critical features for segmenting polyps from endoscopic imagery by this model demonstrated a significant advancement in AI's capability to support highly detailed and nuanced medical diagnostics.

Due to their ability to capture long range dependencies with inbuilt self-attention mechanisms, Transformer models are replacing the conventional segmentation models based on CNNs. Nevertheless, the polyp segmentation model such as the ColonFormer, SSFormer and FCN-Transformer are found to be very complex, with additional attention and feature fusion mechanisms. Further, these models are less adaptive to the shape and size of polyps due to the linear convolutional kernels. Deformable convolutions^51^ which can adaptively define the kernels based on the input are demonstrated to generalize well and converge faster with less memory requirements, compared to CNNs. This research addresses the need for developing Transformer models based on adaptive convolutional kernels, which has not been attempted so far.

## Proposed system

**Figure 1:**
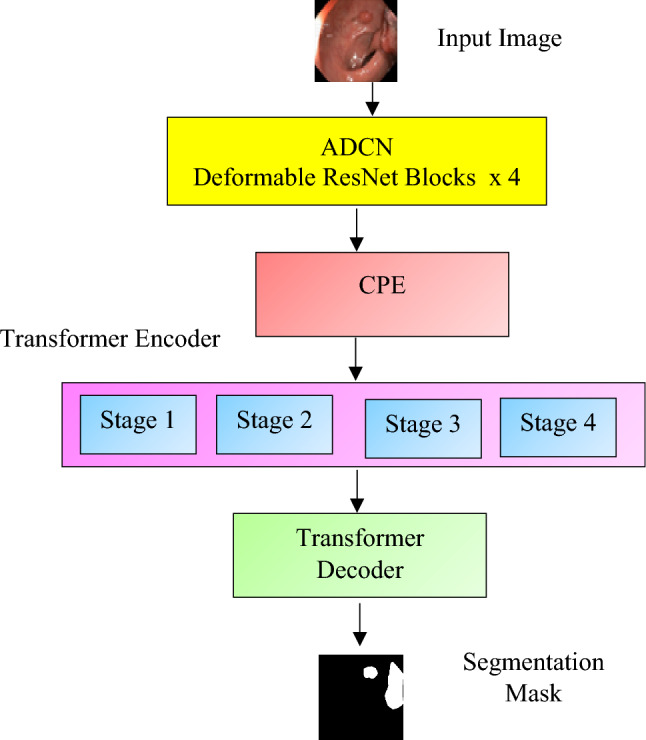
Polyp-ViT segmentation pipeline.

The Polyp-ViT model, proposed herein, is implemented as a dense prediction transformer, featuring an adaptive deformable attention mechanism illustrated in Fig. 1. The architecture encompasses several key components to effectively address the intricacies of colon polyp images. These include the integration of the Adaptive Deformable Convolutional Network (ADCN) for robust feature extraction, the incorporation of Conditional Positional Encoding (CPE) to generate translation and position-invariant feature maps, an encoder for establishing a comprehensive global representation of the image, and a decoder optimized for precise segmentation. The rationale behind this design stems from the imperative to capture both local details and global contextual information within colon polyp images. This dual focus is instrumental in achieving a high level of segmentation accuracy. The Adaptive Deformable Convolution layers are strategically embedded within the Transformer encoder, endowing the model with the adaptive capability to concentrate on pertinent spatial features. Simultaneously, the CPE mechanism is employed to ensure the model retains a robust understanding of spatial relationships within the image—a challenge commonly encountered by standard transformer models in vision tasks. Furthermore, Polyp-ViT's segmentation performance is augmented by its adept utilization of multi-scale features and the seamless incorporation of detailed positional information. These features collectively render Polyp-ViT exceptionally effective for the challenging task of colon polyp segmentation, showcasing its prowess in capturing nuanced details and contextual nuances essential for accurate segmentation results. The operational workflow of the Polyp-ViT model is detailed below, delineating the specific processes at each layer:

1. *Initial Feature Extraction* The input image is first processed through the Adaptive Deformable Convolutional Network (ADCN), which includes four Deformable ResNet Blocks. This stage is crucial for tailoring the feature extraction to the unique contours and sizes of colon polyps.
2. *Positional Encoding* Subsequently, the Conditional Positional Encoding (CPE) module imparts critical positional information to the features. This step is essential to equip the Transformer, which inherently lacks spatial inductive biases, with the necessary spatial awareness.
3. *Feature Abstraction and Contextualization* The features are then propagated through four distinct stages within the Transformer encoder. Each stage is meticulously designed to increment the level of feature abstraction, enriching the global contextual understanding of the image.
4. *Global Representation Refinement* As the features progress through the encoder stages, they undergo a comprehensive refinement process, ensuring a rich global representation of the image is formed, which is vital for accurate segmentation.
5. *Segmentation Mask Reconstruction* In the final phase, the Transformer decoder takes over, translating the globally encoded features back into the spatial domain. This results in the generation of a precise segmentation mask, distinctly outlining the polyp against the surrounding tissue.

The individual components of the model are described in the following subsections.

## Adaptive deformable convolutional network

Given an image $I$, deformable convolution is a spatial-wise attention module that modifies the weights of convolutional layers to focus on the most relevant region^52^. The core idea of deformable convolution is to select optimal sampling points from $I$ and use a trainable kernel to perform convolution and cover the target region. Unlike the adaptive deformable approach which correlates the spatial and channel information, the mechanism used in this research deploys trainable kernels which adapt themselves to the input. The coefficients of the trainable kernel are obtained as in Eq. (1) where ${k}$ is the trainable kernel and ${\phi }_{mn}$ is the attention map.

**Figure 2:**
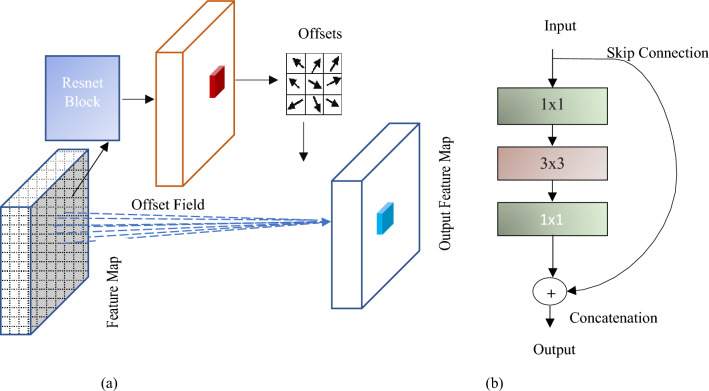
(**a**) Adaptive Deformable convolutional network, (**b**) ResNet block.

The ADCN used in this research is illustrated with Fig. 2. Initially, $k$ is assumed as an all-ones matrix to cover the whole image. and will be gradually updated as the training progresses as in (2), where $H$ and $W$ are the height and width of the attention map ${\phi }_{mn}$. The attention map is captured from the final convolutional layer of the ResNet block.

1 $$\begin{array}{c}{k}_{ij}(x,y)=\sum_{m=0}^{M-1} \sum_{n=0}^{N-1} {k}_{ij}^{mn}\times {\phi }_{mn}(x,y)\end{array}$$

2 $$\begin{array}{c}\begin{array}{c}{\stackrel{\phantom{a}}{k}}_{ij}^{ }(x,y)=\frac{1}{h\times w}\sum_{h=0}^{H-1} \sum_{w=0}^{W-1} {k}_{ij}^{mn}\times {\phi }_{mn}(x,y)\end{array}\end{array}$$

The weights of the convolutional layer are modified to be consistent with the weights of the deformable convolution. The changes in weights are computed as in Eq. (3) and the elements of the kernel are updated as in (4).

3 $$\begin{array}{c}\Delta {W}_{ij}=\sum_{mn} {k}_{ij}^{mn}\times {\phi }_{mn}(x,y)\times \frac{\partial {\phi }_{mn}(x,y)}{\partial x}\times \frac{\partial {\phi }_{mn}(x,y)}{\partial y}\end{array}$$

4 $$\begin{array}{c}{k}_{ij}^{mn}={k}_{ij}^{mn}-\frac{\partial {k}_{ij}^{mn}}{\partial {W}_{ij}}\times \Delta {W}_{ij}\end{array}$$

The offsets are computed based on the premise that the attention map is centered at the center of the target region. The offsets for selecting the sampling points from $I$ are computed from the ${\phi }_{mn}$ as in Eqs. (5) and (6).

5 $$\begin{array}{c}{u}_{i}=\frac{1}{2}\sum_{m=0}^{M-1} \sum_{n=0}^{N-1} {\phi }_{mn}(x,y)\times (m-i)\end{array}$$

6 $$\begin{array}{c}{v}_{j}=\frac{1}{2}\sum_{m=0}^{M-1} \sum_{n=0}^{N-1} {\phi }_{mn}(x,y)\times (n-j)\end{array}$$

The deformable convolution with the trainable kernel is performed by convolving the input image $I$ with the trainable kernel $k$ using the offsets ${u}_{i}$ and ${v}_{j}$ as in (7).

7 $$\begin{array}{c}\widetilde{I}(x,y)=\sum_{m=0}^{M-1} {\stackrel{\phantom{a}}{k}}_{ij}(x,y)\times I(x+{u}_{i},y+{v}_{j})\end{array}$$

## Conditional position encoding mechanism

**Figure 3:**
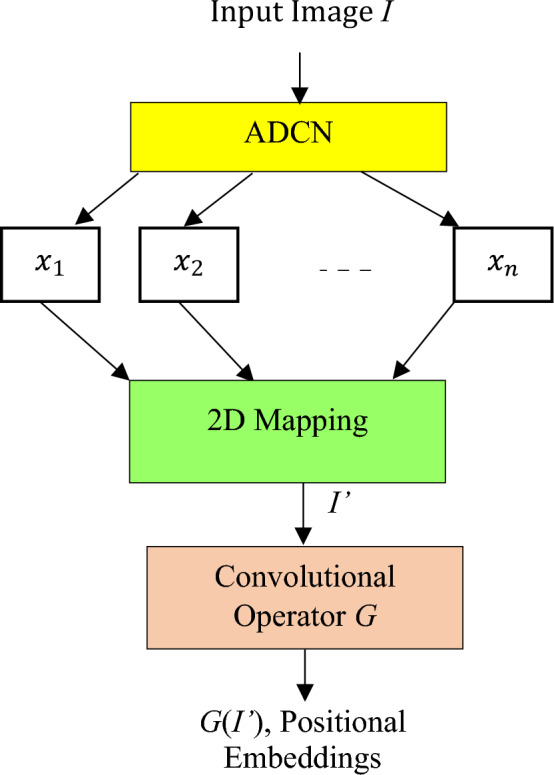
Conditional positioning encoding.

Initially, the feature map is extracted from the input image with an ADCN, and fixed size non-overlapping patches are generated from the feature map. These patches are subjected to conditional positional embedding, using a Position Encoding Generator (PEG) to construct the position embeddings using a convolution operator as shown in Fig. 3.

To apply the convolutional operator which is usually a function $G$, the set of patch sequences $X=\{{x}_{1},{x}_{2},\dots ,{x}_{n}\}$, where $n$ is the number of patches, is mapped to a feature map ${I}{\prime}$ in the 2D space.

## Polyp-ViT encoder

**Figure 4:**
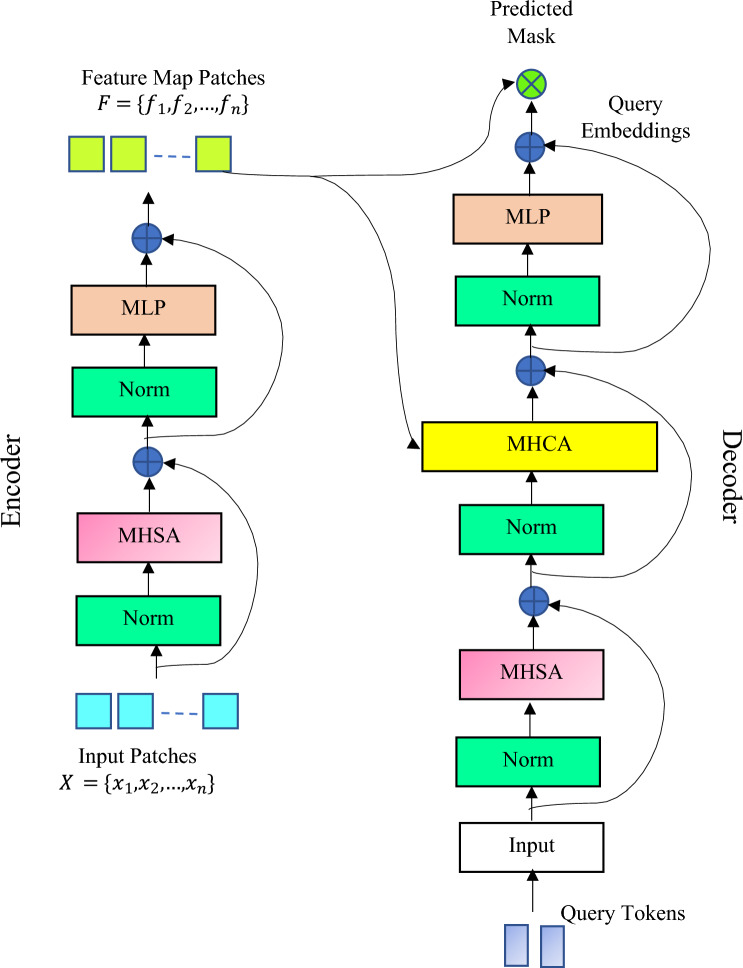
Polyp-ViT encoder–decoder architecture.

The schematic of the proposed Polyp-ViT is given in Fig. 4. The encoder of Polyp-ViT is implemented with four Transformer blocks, each comprising a Multi Head Self Attention (MHSA) and Multilayer Perceptron (MLP) block each preceded by a normalization layer. There are two residual connections one connecting the input to the output of the MHSA, and the other connecting the outputs of the MHSA and that of the MLP. Each Transformer block is connected to the next one and the out of the final block is given as input to the decoder. The patches extracted from the feature maps are concatenated with the positional embeddings and given as input to the first normalization layer. Layer Normalization is an approach used in ViTs to improve the training accuracy by smoothening the gradients. For a given input vector $v$, the Layer Norm (LN) is given as in Eq. (8), where $\gamma$, $\mu$, $\sigma$ and $\beta$ are the scale, mean, standard deviation and bias parameters. These parameters influence the learning ability of the model.

8 $$LN\left(v\right)=\gamma \frac{v-\mu }{\sigma }+\beta$$

The set of normalized patches generated by applying $LN(\cdot )$ on each ${x}_{i}$ is ${X}_{N}=\{{x}_{N1},{x}_{N2},\dots ,{x}_{Nn}\}$. Each normalized patch added with the embedding $\{{x}_{Ni};{e}_{i}\}$ is given as input to the MHSA which is transformed into three vectors viz. Query $Q$, Key $K$ and Value *V* employing three weight matrices ${{W}^{Q}}$, ${{W}^{K}}$ and ${{W}^{Q}}$. The attention score $AS$ is computed from $({Q}_{i},{K}_{i},{V}_{i}$) as in Eq. (9), where ${d}_{k}$ is the dimension of $K$. The MHSA mechanism allows the Transformer to learn semantic features from each patch suitable for image recognition, classification, and segmentation tasks.

9 $$AS=softmax\frac{({Q}_{i} {K}_{i}^{T})}{\sqrt[ ]{{d}_{k}}}{V}_{i}$$

The MLP is implemented as a classification head with a Gaussian Error Linear Unit (GELU) activation function which is differentiable and permits small negative gradients. It allows the MLP to learn the features from the semantic features learned at the MHSA. The residual connections around the MHSA and MLP enable direct flow of the gradients into the network. The mathematical representation of the functions carried out at the Transformer encoder are given in Eqs. (10)–(11).

10 $${Z}_{1}=MHSA\left({x}_{Ni};{e}_{i}\right)+\left({x}_{i};{e}_{i}\right) \forall i=1..n$$

11 $${Z}_{2}=MLP({Z}_{1})+{Z}_{1}$$

The final Transformer block provides a set of image features $F=\{{f}_{1},{f}_{2},\dots ,{f}_{n}\}$ which is given as input to the decoder.

## Polyp-ViT decoder

The decoder of the Polyp-ViT comprises a MHSA, Encoder-Decoder or Multi Head Cross Attention (MHCA) and MLP blocks, LN blocks and residual connections as in Fig. 4. Generally, the decoder takes a series of query tokens as input, one for each class. In this research, there are two classes, Polyp and Background represented with a set of query tokens $T=$ $\{{T}_{1},{T}_{2}$}. The MHSA accepts the learnable query tokens corresponding to these classes as inputs and learns the relationships between them. The Encoder-Decoder attention block takes the features ${f}_{i}$ for each patch *i* and constructs the cross attention map ${m}_{i}$ by learning the relationship between the features and the query tokens. The MLP block constructs the query embeddings ${qe}_{i}$ from each ${m}_{i}$. The segmentation mask is constructed by pixel-wise multiplication of the feature patches and the query embeddings. The functions of the decoder are described with Eqs. (12)–(15).

12 $${Z}_{3}=MHSA(LN\left(T\right))$$

13 $${Z}_{4}=MHCA(LN\left({Z}_{3}\right), F)+{Z}_{3}$$

14 $${Z}_{5}=MLP(LN\left({Z}_{4}\right)+{Z}_{4}$$

15 $$\text{Mask=F}\otimes {\text{Z}}_{5}$$

Given a training dataset $D={\{({x}_{i},{y}_{i})\}}_{i=1}^{N}$, the training loss is computed as in (16) where each ${x}_{i}$ is an input image and ${y}_{i}$ is a ground truth label, where $\theta$ is the set of the parameters of the network, $C$ is the number of classes and $P({y}_{i}=j|{x}_{i},\theta ))$ is the probability of the Polyp class label ${{y}_{i}}=j$ given the input ${x}_{i}$ and the parameters $\theta$.

16 $$\begin{array}{c}L(D,\theta )=\frac{1}{N}\sum_{i=1}^{N}[\sum_{j=1}^{C}{\text{log}}(P(y{ }_{i}=j|{x}_{i},\theta ))]\end{array}$$

The model is trained using Adam optimizer with a learning rate of 0.001. The learning rate is reduced by a factor of 0.01 every 20 epochs. The training process can be further accelerated by introducing the channel dropout at the output of the encoder or decoder. This makes the network robust against overfitting by preventing the activations of neurons from saturating during training. The dropout threshold is set based on the variance of the query tokens and the learning ability of the model with respect to the number of training epochs.

## Experimental works and discussion

This section presents the empirical results on the performance of the Polyp-ViT model, evaluated on the Kvasir-seg and CVC-Clinic DB datasets. The results include visual representation of the segmentation masks, objective metrics and comparisons with state-of-the-art models. Further, ablation study of Polyp-ViT model demonstrates the efficacy of the model.

### Datasets and implementation details

The Polyp-ViT is trained and tested on the publicly available Kvasir-seg and CVC-ClinicDB datasets. The Kvasir-seg dataset comprises 1000 colonoscopy images annotated with ground truth labels. The images and labels appear in varying dimensions such as 1920 × 1072, 1214 × 1019, 401 × 415, 332 × 487 in JPG format. The CVC-ClinicDB dataset consists of 612 images and labels of uniform dimension 384 × 288 in both PNG and TIF formats. The datasets are augmented by applying affine transformations on the images to construct the training and testing subsets. After augmentation, the Kvasir-seg dataset consists of 1000 images in the training and testing datasets, and the ClinicDB dataset has 612 images in the training and testing datasets. For the sake of training and testing the Polyp-ViT network, all the images and labels are resized to 256 × 256 and represented in JPG and PNG formats respectively.

The Polyp-ViT is implemented with the Deep Learning and Image Processing tool boxes in Matlab 2022a, and trained and tested in a i7 processor, equipped with 32 GB DDR4 RAM accelerated by a NVIDIA GeForce GTX1060 3 GB Graphics card. The training datasets are apportioned in the ratio 80:20 for training and validation. Table 1 gives the distribution of the dataset.

**Table 1:** Distribution of dataset.

Dataset	No. of images in training dataset	No. of images in testing dataset	No. of images in validation dataset
Kvasir-seg	800	1000	200
CVC-ClinicDB	490	612	122

### Performance evaluation

The performance of Polyp-ViT is evaluated with the accuracy, specificity $(Sp)$, sensitivity $(Sn)$ and precision $(Pn)$ metrics based on the True Positive (TP), True Negative (TN), False Positive (FP) and False Negative (FN) values. These metrics are defined in Eqs. (17)–(20). Accuracy refers to the number of correctly predicted pixels in the segmentation mask $M$, out of the total number of pixels in the ground truth $G$.

17 $$Accuracy=\frac{(TP+TN)}{\left(TP+TN+FP+FN\right)}$$

Recall or sensitivity is the total number of correctly predicted positive pixels of $M$ out of the total number of positive pixels of $G$ as in (18). Specificity is the of negative(background) pixels correctly identified in $M$ out of the total number of negative samples as in Eq. (19).

18 $$Sensitivity =\frac{TP}{\left(TP+FN\right)}$$

19 $$Specificity=\frac{TN}{\left(TN+FP\right)}$$

Precision is a measure of the correctly identified positive pixels in $M$ out of the total number of positive pixels predicted by the model as in (20).

20 $$Precision=\frac{TP}{\left(TP+FP\right)}$$

Further, the Dice (F1 Score) and weighted IoU metrics are also used. Dice is a measure of the overlap between the ground truth $G$ and the segmentation mask $M$ as in (21), which ranges from 0 to 1. On perfect segmentation, Dice value is around 1.

21 $$Dice=\frac{2\left|M\cap G\right|}{\left|M\right|+\left|G\right|}$$

IoU evaluates the number of correctly predicted pixels in the segmented mask $M$ compared to the ground truth label $G$ as in (22). The weighted IoU is the mean of the IoU of each target label weighted by the number of pixels in the label. This metric is useful to evaluate the models tested with imbalanced datasets. The segmentation results are presented in Table 2 for the two datasets.

**Table 2:** Segmentation performance metrics.

Dataset	Accuracy	Sensitivity	Specificity	Precision	Weighted IoU	Dice
Kvasir-SEG	0.9891 ± 0.01	0.9862 ± 0.04	0.9865 ± 0.60	0.9827 ± 0.31	0.9871 ± 0.34	0.9871 ± 0.79
CVC-Clinic DB	0.9875 ± 0.71	0.9870 ± 0.01	0.9891 ± 0.01	0.9817 ± 0.01	0.9810 ± 0.18	0.9887 ± 0.69

22 $$IoU=\frac{\left|M\cap G\right|}{\left|M\cup G\right|}$$

**Figure 5:**
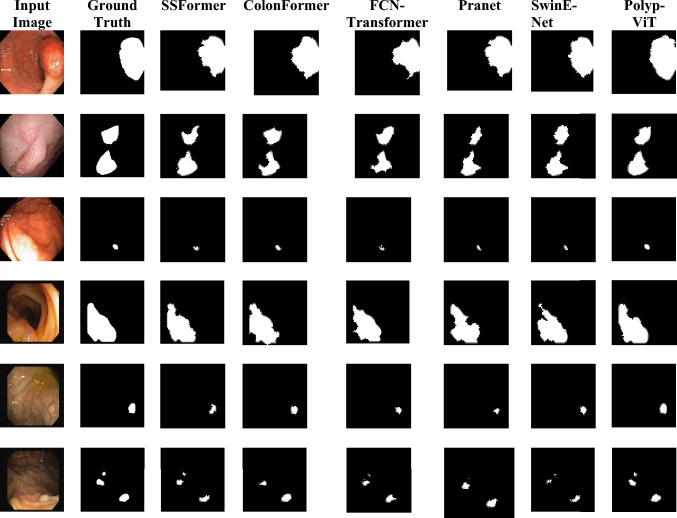
Segmentation results row (1–3) Kvasir-SEG row (4–6) CVC-clinic DB.

Visual illustrations of the input images, ground truths and segmentation masks are given in Fig. 5 for the Polyp-ViT and other transformer based segmentation models. It is seen that the masks predicted by Polyp-ViT are similar to the ground truth.

In this line, the objective performance metrics are compared for the above models in Tables 3 and 4 for the Kvasir-SEG and CVC-Clinic DB datasets respectively. The results are obtained by testing the models with the same test dataset under fivefold cross validation for a fair comparison, and the mean of the metrics are presented. The best values are highlighted in red bold fonts and the second best values are shown in bold blue. It is seen that the of the outputs of the models are consistent for the two datasets, the best performances achieved by Polyp-ViT, demonstrating a significant performance gain. These results align with the visual segmentation masks predicted by Polyp-ViT shown in Fig. 5.

**Table 3:** Segmentation performance—Kvasir-seg dataset.

Model	Metrics					
	mAccuracy	mSensitivity	mSpecificity	mPrecision	mDice	mIoU
Polyp-pvt^35^ (2021)	0.8128 ± 0.90	0.8046 ± 0.19	0.7973 ± 0.25	0.8022 ± 0.15	0.8036 ± 0.91	0.7965 ± 0.37
ColonFormer^17^ (2022)	0.8341 ± 0.23	0.8257 ± 0.28	0.8182 ± 0.09	0.8232 ± 0.29	0.8267 ± 0.61	0.8174 ± 0.84
SSFormer^18^ (2022)	0.8762 ± 0.13	0.8674 ± 0.09	0.8595 ± 0.37	0.8648 ± 0.45	0.8619 ± 0.25	0.8586 ± 0.17
FCN-Transformer^19^ (2022)	0.9395 ± 0.01	0.9302 ± 0.74	0.9217 ± 0.49	0.9274 ± 0.51	0.9251 ± 0.17	0.9208 ± 0.21
SwinE-Net^36^ (2022)	0.7849 ± 0.61	0.7770 ± 0.14	0.7699 ± 0.79	0.7746 ± 0.13	0.7663 ± 0.53	0.7692 ± 0.62
PENet^39^ (2022)	0.9151 ± 0.08	0.9126 ± 0.14	0.9192 ± 0.11	0.9102 ± 0.14	0.9383 ± 0.01	0.8901 ± 0.11
Fu-TransHNet^41^ (2023)	0.9125 ± 0.26	0.9101 ± 0.61	0.9115 ± 0.37	0.9095 ± 0.75	0.9089 ± 0.11	0.9102 ± 0.42
CAFE-Net^50^ (2024)	0.9202 ± 0.33	0.9213 ± 0.06	0.9196 ± 0.08	0.9182 ± 0.53	0.9185 ± 0.19	0.9189 ± 0.25
Polyp-ViT (proposed)	0.9891 ± 0.01	0.9862 ± 0.04	0.9865 ± 0.60	0.9827 ± 0.31	0.9871 ± 0.79	0.9889 ± 0.34

**Table 4:** Segmentation performance—CVC-clinic DB dataset.

Model	Metrics					
	mAccuracy	mSensitivity	mSpecificity	mPrecision	mDice	mIoU
Polyp-pvt^35^ (2021)	0.8118 ± 0.10	0.8091 ± 0.25	0.7893 ± 1.02	0.8115 ± 0.91	0.8128 ± 0.19	0.7831 ± 0.41
ColonFormer^17^ (2022)	0.8350 ± 0.41	0.8179 ± 0.44	0.8202 ± 0.28	0.8174 ± 0.07	0.8203 ± 0.63	0.8159 ± 0.12
SSFormer^18^ (2022)	0.8622 ± 0.24	0.8574 ± 0.01	0.8329 ± 0.14	0.8571 ± 0.56	0.8506 ± 0.82	0.8359 ± 0.27
FCN-Transformer^19^ (2022)	0.9250 ± 0.13	0.9291 ± 0.14	0.9119 ± 0.49	0.9101 ± 0.91	0.8964 ± 0.53	0.9195 ± 0.11
SwinE-Net^36^ (2022)	0.7790 ± 0.12	0.7781 ± 0.51	0.7849 ± 0.71	0.7598 ± 0.90	0.7810 ± 0.04	0.7641 ± 0.55
PENet^39^ (2022)	0.9011 ± 0.08	0.8912 ± 0.04	0.9027 ± 0.12	0.8926 ± 0.24	0.9178 ± 0.21	0.8681 ± 0.17
Fu-TransHNet^41^ (2023)	0.9201 ± 0.17	0.9196 ± 0.11	0.9181 ± 0.03	0.9191 ± 0.08	0.9183 ± 0.45	0.9176 ± 0.38
CAFE-Net^50^ (2024)	0.9276 ± 0.47	0.9259 ± 0.20	0.9248 ± 0.14	0.9196 ± 0.23	0.9189 ± 0.61	0.9176 ± 0.26
Polyp-ViT (proposed)	0.9875 ± 0.71	0.9870 ± 0.01	0.9891 ± 0.01	0.9817 ± 0.01	0.9887 ± 0.69	0.9810 ± 0.18

### Ablation experiments

In this research, the ablation experiment is performed to evaluate the performance of the model without the deformable convolution operator. This study is carried out to establish the significance of the adaptive convolution operation performed at the ResNet50 blocks and the CPE. The performance of Polyp-ViT is evaluated with the feature maps constructed with the ResNet50. The experiments are conducted on the test datasets by fivefold cross validation and the mean values of the metrics are presented in Table 5. The best metrics for each of the datasets are shown in bold faces.

**Table 5:** Performance metrics on ablation studies. Significant values are in bold.

Dataset	Metrics					
	mAccuracy	mSensitivity	mSpecificity	mPrecision	mDice	mIoU
Kvasir-SEG (ResNet50 + ADCN + CPE)	**0.9489**	**0.9395**	**0.9309**	**0.9367**	**0.9243**	**0.9300**
Kvasir-SEG (ResNet50)	0.8104	0.8023	0.7950	0.7999	0.7893	0.7942
CVC-clinic DB (ResNet50 + ADCN + CPE)	0.9259	0.9165	0.9082	0.9138	0.9176	0.9073
CVC-clinic DB (ResNet50)	0.7870	0.7790	0.7720	0.7767	0.7800	0.7712

**Figure 6:**
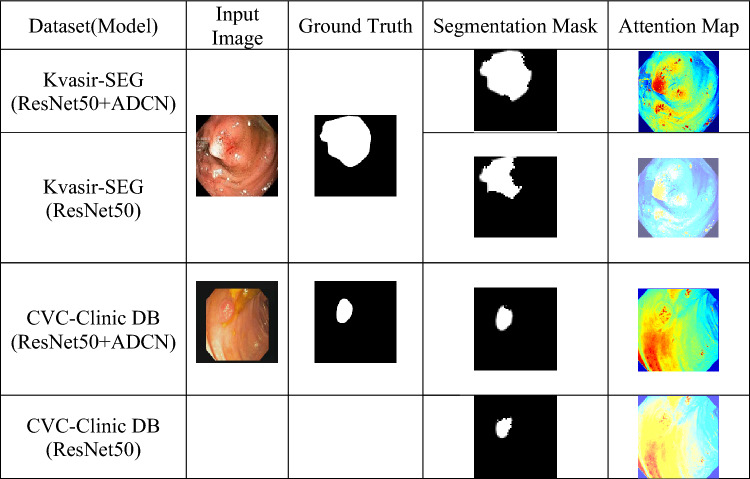
Segmentation masks and attention maps under ablation study.

It is seen that there is a degradation in the segmentation performance metrics roughly by 10% for both the datasets. Further, the segmentation masks are shown in Fig. 6, to visualize the performance of the model in the absence of deformable convolution. It is seen that the attention maps captured from the model with the ADCN are highly expressive, compared to that of the ResNet50 blocks, and the masks predicted from these maps closely match the ground truths.

## Discussion

The experimental results show that Polyp-ViT outperforms the state-of-the-art models in objective metrics and the predicted masks are quite similar to the ground truth masks. It is seen from Tables 2 and 3 that the FCN-Transformer which is a hybrid model demonstrates the best performances next to Polyp-ViT. Compared to other state-of-the-art models. As mentioned in Section “Related works”, FCN-Transformer is based on SSFormer and employs two separate branches for extraction of coarse and fine features. Further, it also employs an exclusive fusion mechanism to fuse these features. Comparatively, Polyp-ViT follows the general ViT architecture and it is enhanced with the ADCN mechanism for sample point selection adaptively with learnable kernels, and a CPE mechanism to construct the positional embeddings.

A detailed analysis of the representative polyp segmentation models with respect to their design characteristics and performance measures is presented in Table 6 to highlight the significance of the Polyp-ViT model. This table comparing various polyp segmentation models across the Kvasir-seg and ClinicDB datasets highlights a spectrum of approaches, showcasing the trade-off between innovation, performance, and efficiency. Polyp-ViT emerges as a standout for its exceptional accuracy and low mean execution time, emphasizing its advanced architecture that incorporates Adaptive Deformable Convolution Network (ADCN) and Conditional Positional Encodings (CPE), despite its reliance on ResNet blocks and limited evaluation on polyp sizes. In contrast, models like SwinE-Net and PENet, despite their innovative use of EfficientNet with Swin Transformer and Transformer Inception with Local-Detail Augmentation modules respectively, face challenges in computational demands and generalization, reflecting the broader challenge in the field: balancing complex architectures' capabilities with their practical applicability and generalization across diverse clinical environments. This analysis underscores the importance of optimizing model architectures not only for segmentation accuracy but also for computational efficiency and adaptability, steering future advancements in medical imaging towards more versatile and efficient solutions.

**Table 6:** Comparison of model performance and characteristics.

Model	Dataset	Model accuracy	Mean execution time/image (ms)	Advantages	Limitations
Polyp-pvt^35^ (2021)	Kvasir-seg	0.8128 ± 0.90	349	Employs a PVT with modules for semantic capture, attention to polyp cues, and feature aggregation, effectively enhancing polyp segmentation​​	Sensitive to light, shadow, and reflective points; requires multiple modules for feature localization and aggregation, which may affect generalization across diverse conditions
	ClinicDB	0.8118 ± 0.10	376		
ColonFormer^17^ (2022)	Kvasir-seg	0.8341 ± 0.23	293	Combines a hierarchical Transformer encoder with a pyramid CNN decoder, utilizing Mix Transformer and Pyramid Pooling Model for enhanced feature representation and segmentation	Complex model with repeated feature extraction and segmentation map creation. This complexity hinders generalization abilities
	ClinicDB	0.8350 ± 0.41	307		
SSFormer^18^ (2022)	Kvasir-seg	0.8762 ± 0.13	285	Employs a PVTv2 pyramid vision transformer for encoding, integrating a convolutional feedforward network and attention layer for enhanced segmentation	This model features hybrid architecture complexity. Refinement is needed for improved segmentation, which may limit adaptability to new datasets
	ClinicDB	0.8622 ± 0.24	272		
FCN-Transformer^19^ (2022)	Kvasir-seg	0.9395 ± 0.01	115	Integrates a Transformer Branch with a Fully Convolutional Branch, employing residual blocks and SiLU activation for effective feature fusion and segmentation	This model is implemented with a PVTv2 whose architecture is complex compared to standard ViTs. Explicit attention and fusion mechanisms in decoder may not generalize well across varied datasets
	ClinicDB	0.9250 ± 0.13	147		
SwinE-Net^36^ (2022)	Kvasir-seg	0.7849 ± 0.61	105	merges EfficientNet and Swin Transformer for polyp segmentation, utilizing dilation convolutional blocks and a multi-feature aggregation block for enhanced feature extraction and segmentation accuracy	This model follows a hybrid architecture comprising a Transformer and a fully convolutional network. The transformer is an enhanced version of the SSFormer adding complexity resulting in poor generalization
	ClinicDB	0.7790 ± 0.12	112		
PENet^39^ (2022)	Kvasir-seg	0.9151 ± 0.08	72	Transformer Inception and Local-Detail Augmentation modules, enriching features with long-range information and enhancing details for efficient and accurate polyp segmentation​​	This model requires substantial computational resources due to the utilization of parallel computation-based modules, which limits its practical applicability in resource-constrained environments. The model does not generalize well to all clinical environments due to parallel computation requirements
	ClinicDB	0.9011 ± 0.08	89		
Fu-TransHNet^41^ (2023)	Kvasir-seg	0.9125 ± 0.26	54	Fusion of Transformer and CNN branches enhances both global and local feature learning, potentially improving adaptability and precision in polyp segmentation	Potential integration complexities between Transformer and CNN features, and increased computational demands, which may impact efficiency and applicability in diverse settings
	ClinicDB	0.9201 ± 0.17	62		
CAFE-Net^50^ (2024)	Kvasir-seg	0.9202 ± 0.33	43	Leverages cross-attention mechanisms and feature exploration networks, indicating a potentially significant advancement in detailed and nuanced medical diagnostics	This model features advanced cross-attention mechanisms and feature exploration networks, potentially leading to higher computational costs and adaptation challenges to variable data sets
	ClinicDB	0.9276 ± 0.47	47		
Polyp-ViT (proposed)	Kvasir-seg	0.9891 ± 0.01	23	Conventional ViT structure is enhanced with ADCN and CPE mechanisms. This model is less rugged compared to the representative polyp segmentation models	This model utilizes ResNet blocks within its Transformer encoder despite the availability of various pre-trained models for feature extraction. The model can be evaluated with such models in the encoder as well
	ClinicDB	0.9875 ± 0.71	21		

This analysis reveals that best segmentation performances can be obtained with simple Transformer models harnessing the inbuilt attention mechanisms, by adaptively selecting the sample points for feature extraction and adaptive position embedding. Further, the positional embeddings are conditionally selected with a convolutional operator applied on the feature maps extracted by adaptive deconvolution. In contrast to the absolute positional embedding in prior models, the CPE mechanism used in this research is conditioned on the local neigbourhood. This is again testified with the ablation study in which the model is evaluated without the ADCN and CPE.

There are two limitations of Polyp-ViT which are worth discussing. The first is the usage of ResNet blocks in the Transformer encoder, in spite of the availability of several pre-trained models which can be used in feature extraction. The ResNet is chosen considering its merits over other networks such as low training error and lack of vanishing gradient problem. Empirical evaluation of Polyp-ViT with other networks can provide a more insightful selection of the backbone network for feature extraction. The second limitation is the lack of evaluation of the performance of Polyp-ViT on the segmentation of different sized polyps. Though Polyp-ViT has been evaluated with two distinct datasets, the ability to segment small and very small polyps has not been testified. Exclusive annotated datasets need to be developed to address this requirement.

Despite its limitations, the Polyp-ViT model presents a groundbreaking Transformer-based framework for polyp segmentation, showcasing potential applicability to a range of pathologies. This framework is designed with flexibility at its core, allowing for modifications tailored to specific medical segmentation tasks. By facilitating task-specific adjustments within the Polyp-ViT architecture, the model not only excels in its current domain but also sets a foundation for future innovations across medical imaging, promising to revolutionize the approach to diagnosing and understanding various diseases. This adaptability underscores the model's significant contribution to advancing AI in healthcare, offering a versatile tool for enhancing accuracy and efficiency in medical diagnoses.

## Clinical relevance

This section outlines the practical implications of deploying Polyp-ViT in clinical settings. It discusses how the model can be integrated into clinical workflows, necessary adjustments for real-world application, challenges in clinical deployment including regulatory hurdles and training needs, and the expected benefits on patient care and diagnostic efficiency. The aim is to highlight the model's potential to enhance early detection of colon polyps, thereby improving patient outcomes and streamlining the diagnostic process in gastroenterology.

### Integration into clinical workflows

To integrate the model into clinical workflows, it can be embedded in diagnostic tools used in colonoscopy suites, providing real-time polyp detection and segmentation. This approach supports decision-making during procedures, enabling immediate biopsy or polypectomy of suspicious lesions. Integration requires seamless connectivity with Electronic Health Records (EHRs) and imaging systems, ensuring that polyp detections are documented and accessible for review and further analysis.

### Modifications for clinical usage

Adapting the model for clinical use involves calibrating it to match the specific characteristics of imaging equipment across different healthcare providers, ensuring consistent performance. This might include tuning the model to work with various endoscopic video resolutions and lighting conditions. User feedback mechanisms could be incorporated to refine model predictions based on clinician input, enhancing accuracy over time through continuous learning.

### Challenges in clinical implementation

Securing regulatory approval presents a significant hurdle, necessitating rigorous validation studies to demonstrate the model's safety and efficacy. Data privacy and security measures must comply with healthcare regulations such as Health Insurance Portability and Accountability Act (HIPAA) in the United States. Training programs for clinicians and technical staff are essential to ensure they are proficient in using the new system and interpreting its outputs within the clinical context.

### Impact on clinical outcomes

The model's integration into clinical practice is expected to improve early detection rates of colon polyps, leading to proactive management of colorectal cancer risks. By providing high-precision polyp detection, the model can enhance the diagnostic accuracy, potentially reducing the incidence of missed lesions. This improvement in diagnostic efficiency could lead to better patient outcomes, lessening the burden of colorectal cancer through early intervention and reducing the healthcare system's reliance on costly, invasive diagnostic procedures.

## Conclusion

This research presents a novel model for Polyp segmentation from colonoscopy images. The proposed Polyp-ViT is a dense prediction Transformer which is built from the conventional ViT architecture by enhancing it with an adaptive mechanism for feature extraction and positional embedding. The ADCN is a deformable convolutional mechanism which employs learnable kernels to select the sampling points in an image to extract the features with long-range dependencies. The CPE is an adaptive position embedding mechanism capable to learning the local image contexts with a convolutional operator. Experimental results show that Polyp-ViT achieves mean Dice values of 0.9871 ± 0.79 and 0.9887 ± 0.69 on the Kvasir-seg and CVC-Clinic DB Datasets respectively. This model is a promising tool for early diagnosis of polyps towards reducing the mortalities and morbidities due to CRC. This model can be integrated with clinical protocols in the diagnosis, staging and severity analysis of the polyps manifesting several organs.

## Data Availability

The datasets generated during and/or analyzed during the current study are publicly available in Jha, D. et al. (2020). Kvasir-SEG: A Segmented Polyp Dataset. In: Ro, Y., et al. MultiMedia Modeling. MMM 2020. Lecture Notes in Computer Science, vol. 11962. Springer, Cham. 10.1007/978-3-030-37734-2_37.
